# Patient‐derived organoid‐immune co‐cultures integrated with multi‐omics reveal immunotherapy resistance mechanisms in urothelial carcinoma

**DOI:** 10.1002/imt2.70130

**Published:** 2026-05-07

**Authors:** Shan Jiang, Yuxuan Song, Yun Peng, Ran Yan, Yunze Niu, Baoqiang Chen, Jiaxing Lin, Jilin Wu, Shixiang Wang, Yiqing Du, Caipeng Qin, Yihan Lin, Tao Xu

**Affiliations:** ^1^ Department of Urology Peking University People's Hospital Beijing China; ^2^ Center for Quantitative Biology and Peking‐Tsinghua Center for Life Sciences, Academy for Advanced Interdisciplinary Studies Peking University Beijing China; ^3^ Beijing Chaoyang Hospital Capital Medical University Beijing China; ^4^ Department of Urology Fuzhou University Affiliated Provincial Hospital Fuzhou China; ^5^ Department of Biomedical Informatics, School of Life Sciences Central South University Changsha China

## Abstract

Immunotherapy resistance presents a formidable challenge in tumor biology. While fibroblast growth factor receptor 3 (FGFR3) serves as a pivotal oncogenic driver in a multitude of cancers, the exploration of its role in immune checkpoint inhibitor (ICI) resistance remains scarce, thus impeding a deeper understanding of the tumor immune microenvironment (TIME) in the era of immunotherapy. Employing patient‐derived urothelial carcinoma (UC) organoids and co‐cultured systems, along with single‐cell RNA sequencing (scRNA‐seq), whole‐exome sequencing (WES), bulk RNA‐seq, and CUT& Tag epigenomics in UC cohorts, we identified and characterized the key downstream mediators of FGFR3. The TIME associated with *FGFR3* mutations exhibited a depletion of NK and CD8^+^ T cells, while simultaneously harboring an accumulation of exhausted effectors, correlating with diminished ICI response. Erdafitinib reprogrammed this “cold” TME into an inflamed state through a novel FGFR3–STAT5–IRF2 signaling cascade. These findings, corroborated by a wealth of evidence, advocate for the combination of FGFR3‐targeted therapy with immunotherapy for UC, bridging critical pre‐clinical and clinical insights.

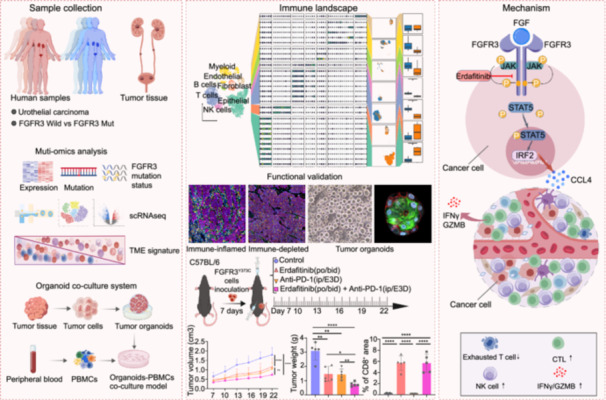

To the Editor,

Urothelial carcinoma (UC), including bladder cancer (BC) and upper tract urothelial carcinoma (UTUC), arises from the urothelium lining the urinary tract [1, 2]. BC ranks as the ninth most common cancer globally, with ~614,000 new cases and ~220,000 deaths in 2022, while UTUC accounts for 5–10% of UC cases and is frequently diagnosed at advanced, muscle‐invasive stages [3, 4].

Fibroblast growth factor receptor 3 (FGFR3) is a key oncogenic driver in UC, linked to reduced immune checkpoint inhibitor (ICI) efficacy [5, 6]. The approval of erdafitinib by the FDA in 2019 for *FGFR3*‐altered metastatic UC marked the beginning of targeted therapies in this disease [7, 8, 9, 10, 11, 12]. *FGFR3* alterations, including common missense mutations such as S249C (48.1%), R248C (9.2%), and Y373C (13.2%), drive oncogenesis in 60–70% of non‐muscle‐invasive BC, 10–20% of muscle‐invasive BC, and 70% of UTUC, promoting ligand‐independent activation and influencing the tumor microenvironment (TME) toward immunosuppression [5, 6, 13, 14, 15]. Despite extensive clinical research on FGFR3, few studies have elucidated its mechanistic role in tumor progression and drug resistance, which has limited our understanding of its impact on the tumor immune microenvironment in the era of immunotherapy. To bridge this gap, advanced pre‐clinical models are needed. Patient‐derived organoid (PDO) models are powerful tools that generally recapitulate the molecular characteristics and heterogeneity of tumor types, and early evidence suggests that screening PDOs can predict patient response to therapy [16, 17, 18, 19].

Using patient‐derived urothelial carcinoma organoids (UCOs) co‐cultured with autologous peripheral blood mononuclear cells (PBMCs), in combination with single‐cell RNA sequencing (scRNA‐seq), whole‐exome sequencing (WES), bulk RNA sequencing (bulk RNA‐seq), multicolor immunofluorescence, and CUT&Tag epigenomics across UC patient cohorts and a C57BL/6 mouse model, we comprehensively mapped the downstream effectors of *FGFR3*.

In *FGFR3*–mutant tumors, the tumor immune microenvironment exhibits depletion of natural killer (NK) cells and CD8⁺ T cells, alongside enrichment of exhausted effector populations, which correlates with poor responses to ICIs. Treatment with erdafitinib reprograms this immunologically “cold” TME into an inflamed state through a novel FGFR3–STAT5–IRF2 signaling cascade, thereby enhancing antigen presentation and promoting lymphocyte trafficking. These mechanistic insights, rigorously validated in both PDO and syngeneic mouse models, provided a strong preclinical rationale for combining FGFR3‐targeted therapy with ICIs in *FGFR3*–mutant UC. The findings effectively bridged critical preclinical evidence with potential clinical translation.

## FGFR3 INHIBITION REVERSES IMMUNE‐DEPLETED MICROENVIRONMENT AND REDUCES T‐CELL EXHAUSTION TO ENHANCE ANTI‐TUMOR IMMUNE RESPONSES

**Figure 1:**
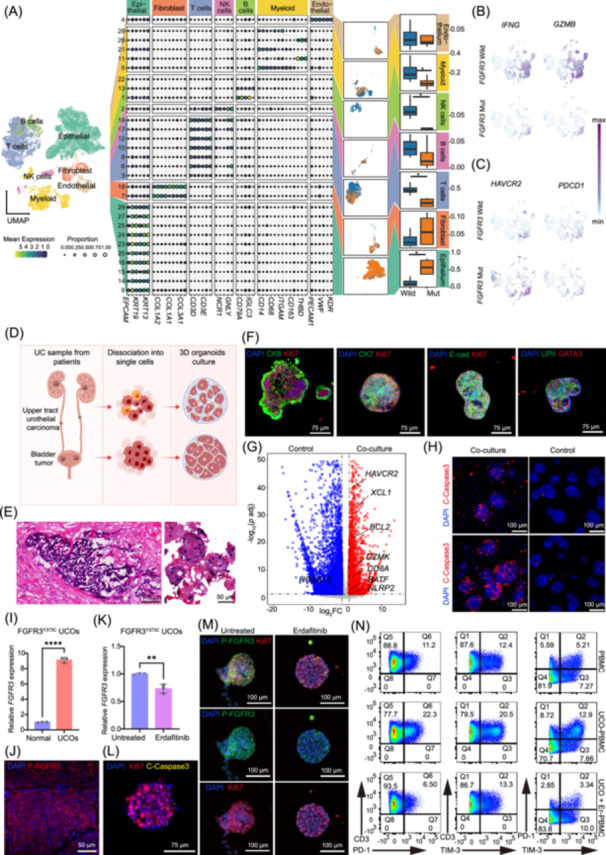
FGFR3 inhibition overcomes immune suppression in *FGFR3*–mutant urothelial carcinoma (UC) co‐culture models. (A) Comparison of cell type distribution and abundance at single‐cell resolution. Left: uniform manifold approximation and projection (UMAP) visualization of all cells, with clusters annotated by canonical cell‐type markers. Middle: individual UMAP plots depicting the distribution of each cell type proportions across *FGFR3* mutation groups. Right: boxplots quantifying differences in cell type proportions across the *FGFR3* mutation groups. (B,C) UMAP visualization of T cells colored by expression levels of *IFNG* and *GZMB* (B), and *HAVCR2* and *PDCD1* (C) across the indicated groups. (D) Schematic overview of the experimental workflow for urothelial carcinoma organoids (UCOs) derivation and culture. Created with BioGDP.com. (E) Representative hematoxylin and eosin (H&E)‐stained sections of UCOs and their corresponding parental tumor tissues. Scale bar: 100 μm (left), 50 μm (right). (F) Immunofluorescence images of UCOs stained for CK8, CK7, GATA3, Ki67, UPII, and E‐cad. Nuclei were counterstained with DAPI (blue). Scale bar: 75 μm. (G) Volcano plot of differential gene expression analysis comparing peripheral blood mononuclear cells (PBMCs) versus UCOs‐PBMCs co‐culture group. (H) Immunofluorescence images of UCOs stained for cleaved caspase3 (C‐Caspase3). Nuclei were counterstained with DAPI (blue). Scale bar: 100 μm. (I) Real‐time qPCR quantification of *FGFR3* expression in UCOs and their parental *FGFR3*–mutant UC tissues. (J) Immunofluorescence images of *FGFR3*–mutant UC sample stained for phosphorylated FGFR3 (P‐FGFR3), Nuclei were counterstained with DAPI (blue). Scale bar: 50 μm. (K) Real‐time qPCR quantification of *FGFR3* expression in UCOs treated with erdafitinib compared to the control. (L) Immunofluorescence images of UCOs stained for cleaved caspase‐3 (C‐Caspase3) following a 24‐h erdafitinib treatment. Nuclei were counterstained with DAPI (blue). Scale bar: 75 μm. (M) Immunofluorescence images of UCOs derived from FGFR3–mutant UC stained for P‐FGFR3 and Ki67 in the presence or absence of erdafitinib. Nuclei were counterstained with DAPI (blue). Scale bar: 100 μm. (N) Flow cytometry analysis of CD3^+^PD‐1^+^, CD3^+^TIM‐3^+^, and PD‐1^+^TIM‐3^+^ T cell proportions in different co‐culture groups. In (I) and (K), asterisks denote statistical significance: *p < 0.05, **p < 0.01, ***p < 0.001, ****p < 0.0001. Wild: wildtype, Mut: mutant, Er: Erdafitinib.

In the UC patient cohort (Table S1), *FGFR3* mutations were associated with an immune‐depleted TME characterized by reduced infiltration of NK cells, cytotoxic CD8^+^ T lymphocytes (CTLs), B cells, and macrophages (Figure 1A,B, Figure S2A), alongside increased T‐cell terminal exhaustion marked by elevated PD‐1 and TIM‐3 expression (Figure 1C, Figure S2B). Analysis of eight patient‐derived tumors (four *FGFR3*–mutant, four wild‐type) via WES (Figure S1A,B) and scRNA‐seq (84,534 cells) revealed higher epithelial cell proportions and *FGFR3* expression in mutant tumors (Figure S1C,D), but significantly lower immune cell fractions, diminished cytotoxicity scores, and heightened exhaustion in T cells (Figure S1E,F), validated spatially by multicolor immunofluorescence (Figure S2C). To model tumor‐immune interactions, patient‐derived UCOs were generated (Figure 1D, Figure S3A), faithfully recapitulating histological features (H&E staining, Figure 1E) and marker expression (CK8, CK7, E‐cad, UPII, GATA3, and Ki67) (Figure 1F). Co‐culture of UCOs with autologous PBMCs (Figure S3B), including three *FGFR3*–mutant UCO lines (Table S2), elicited robust antigen‐specific T‐cell activation. These findings were corroborated by enhanced expression of activation markers *CD103* and *CD69* (Figure S3C, Figure S4A), upregulation of cytotoxicity genes such as *IFNγ* and *GZMB* (Figure 1G), selective cytotoxicity against autologous UCOs (Figure 1H), and enrichment of gene sets associated with T‐cell, B‐cell, and broader leukocyte activation (Figure S3D–F).

Patient‐derived UCOs harboring FGFR3 mutations displayed significantly higher FGFR3 mRNA expression compared to adjacent normal urothelial tissue, preserving the tumor‐associated overexpression phenotype observed in the original clinical samples (Figure 1I, Figure S4B). FGFR3 mutations in UC cause aberrant autophosphorylation and sustained activation of the intracellular tyrosine kinase domain, thereby driving increased levels of phosphorylated FGFR3 (P‐FGFR3) (Figure 1J, Figure S4C). Targeting FGFR3 signaling with erdafitinib in a mutant UCO line (Y373C) effectively inhibited pathway activation (reduced FGFR3 mRNA and P‐FGFR3 levels) (Figure 1K,M) without compromising organoid viability or proliferation (Figure 1L). In co‐cultures, erdafitinib pretreatment significantly decreased proportions of PD‐1^+^, TIM‐3^+^, and double‐positive terminally exhausted T cells, enhancing T‐cell‐mediated cytotoxicity against organoids (Figure 1N, Figure S4D). These findings collectively demonstrate that *FGFR3* mutations drive an immunosuppressive microenvironment through reduced immune infiltration and increased T‐cell exhaustion, and that FGFR3 inhibition effectively reverses T‐cell exhaustion and potentiates anti‐tumor immune responses in UC.

## FGFR3 INHIBITION ENHANCES NK CELL PROPORTION, ACTIVATION, AND TUMOR IMMUNITY VIA STAT5–IRF2‐MEDIATED IFN RESPONSE

**Figure 2:**
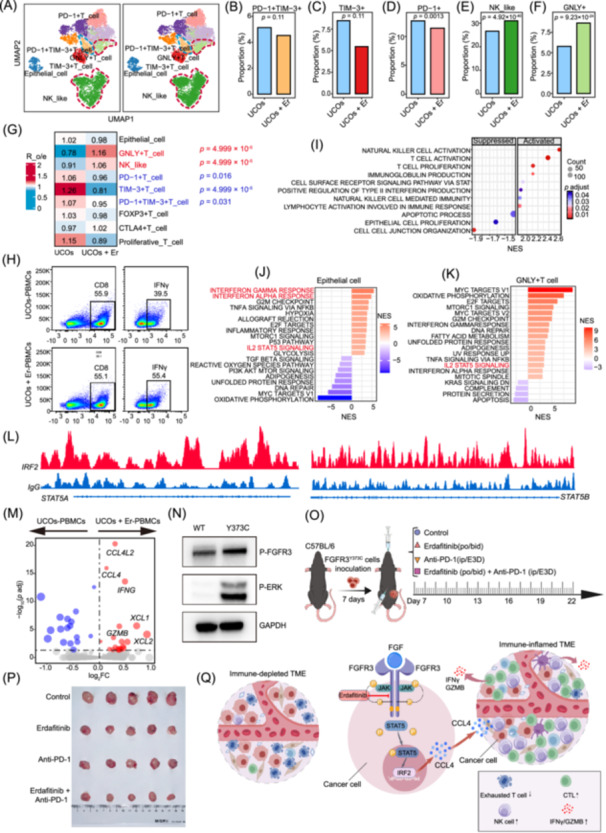
FGFR3 inhibition enhances natural killer (NK) cells activation and recruitment via the STAT5–IRF2 axis in co‐cultures and in vivo models. (A) UMAP plots showing immune cell subtypes identified by unsupervised clustering. (B–F) Bar plots comparing the proportions of immune cell subsets across different co‑culture groups, with a focus on exhausted T cells (PD‑1⁺TIM‑3⁺), NK cells, and GNLY^+^ cytotoxic T cells (CTLs). (G) Heatmap illustrating the tissue‐specific distribution of cellular subpopulations across different co‐culture groups. (H) Flow cytometry analysis of CD8^+^ and CD8^+^IFNγ^+^ T cells in different co‐culture groups. (I) Dot plots summarizing gene set enrichment analysis (GSEA) results, where each point represents an enriched pathway. (J,K) Bar plots summarizing GSEA results of epithelial cells (J) and GNLY^+^ cytotoxic T cells (K). Bar length and color reflect the normalized enrichment score (NES) for each enriched pathway. All enrichment items including the marked one are statistically significant and the adjusted *p* were all < 0.05. (L) CUT& Tag in *FGFR3*–mutant UC cells revealing IRF2 occupancy at the promoters of *STAT5A* and *STAT5B*. (M) Volcano plot of differential gene expression analysis comparing UCOs‐PBMC co‐culture group with the UCOs + Er (erdafitinib) − PBMCs co‐culture group. (N) Western blot analysis of FGFR3 phosphorylation (P‐FGFR3) and MAPK pathway phosphorylation (P‐ERK) across the indicated groups. (O) Schematic of the experimental design for the FGFR3^Y373C^ MB49 tumor‐bearing mouse model. Created with BioGDP.com. (P) Macroscopic images of excised tumors from each group. (Q) Proposed mechanism by which oncogenic FGFR3 signaling shapes an immune‐depleted tumor immune microenvironment (TME) in UC. po/bid: oral administration/twice daily; ip/E3D: intraperitoneal injection/every 3 days.

To investigate the potential mechanisms by which *FGFR3* drives immunosuppression, we examined the effects of pharmacological FGFR3 inhibition with erdafitinib on the TME at the single‐cell level in co‐culture systems (Figure 2A, Figure S5A–C). Erdafitinib treatment significantly reduced the proportions of exhausted T‐cell subsets—including PD‐1⁺, TIM‐3⁺, and PD‐1⁺TIM‐3⁺ double‐positive cells (Figure 2B–D), while substantially increased the abundance of GNLY⁺ cytotoxic T cells (CTLs) and NK‐like cells (Figure 2E,F). This shift was accompanied by upregulated expression of key cytotoxic effectors (*GNLY* and *IFNγ*) and enhanced NK cell‐mediated cytotoxicity (Figure 2G,H), together with evidence of broader T‐cell activation (Figure 2I).

Further analysis identified IRF2 as a consistently upregulated transcription factor in erdafitinib‐treated *FGFR3*–mutant UCOs (Figure S5D); CUT&Tag confirmed IRF2 binding to promoters of key IFN target genes, positioning it as a major regulator of IFN responses downstream of FGFR3 signaling (Figure S5E,F). Notably, erdafitinib also activated the IL2‐STAT5 pathway in GNLY^+^ CTLs and epithelial cells (Figure 2J,K), with *STAT5* expression significantly lower in *FGFR3*–mutant versus wild‐type contexts but markedly upregulated upon FGFR3 inhibition (Figure S6A,B). CUT&Tag profiling of IRF2 revealed prominent promoter binding at *STAT5A* and *STAT5B* loci (Figure 2L), alongside significant enrichment of STAT5 motifs within IRF2 peaks in UC samples and positive expression correlations (Figure S6C and Table S3). Functionally, in co‐culture systems, STAT5 inhibition (STAT5‐IN‐1) suppressed IFN signaling (Figure S6D). Co‐treatment with erdafitinib could reverse suppressed IFN signaling and resulted in elevated IFNγ and GZMB secretion and augmented selective cytotoxicity against autologous UCOs (Figure S6E,F).

Collectively, these findings indicate that FGFR3 inhibition by erdafitinib relieves immunosuppression in *FGFR3*–mutant UC by enhancing pro‐inflammatory IFN signaling in tumor cells through a STAT5–IRF2 transcriptional regulatory axis, thereby promoting anti‐tumor immunity in TME.

## FGFR3 BLOCKADE WITH ERDAFITINIB REPROGRAMS THE TME BY ACTIVATING AND RECRUITING CTLS AND NK CELLS VIA IRF2‐CCL4 SIGNALING AXIS

In addition to promoting the infiltration of CTLs and NK cells into the UC TME, FGFR3 inhibition with erdafitinib also enhanced the activation of NK cells and response to IFNγ in the UCOs‐PBMCs co‐culture model (Figure S7A,B and Table S4). Further investigation revealed significantly elevated expression of chemokines *CCL4L2*, *CCL4*, *XCL1*, and *XCL2* in the erdafitinib‐treated group, as identified by scRNA‐seq, with these chemokines known to facilitate CTLs and NK cells recruitment in solid tumors (Figure 2M). Overexpression of IRF2 in MB49 cells resulted in significant upregulation of *CCL4* at both the RNA and protein levels (Figure S7C,D). CUT&Tag analysis confirmed direct IRF2 binding to the promoters of these chemokines, indicating transcriptional regulation by IRF2 (Figure S7E). In vitro co‐culture assays showed that STAT5 inhibition significantly decreased *IRF2* and *CCL4* RNA expression levels, accompanied by reduced infiltration of CD8⁺ T cells into the organoids (Figure S7F,G). To validate the proposed IRF2‐CCL4 axis in vivo, we engineered an mFGFR3^Y373C^‐expressing MB49 cancer cell line to establish a syngeneic tumor model in C57BL/6 mice (Figure 2N). As anticipated, treatment with erdafitinib increased IRF2 and CCL4 expression, alongside enhanced infiltration of GZMB^+^ CTLs and NK cells, as assessed by multiplex immunofluorescence, consistent with in vitro findings and patient UC tissue analyses (Figure S7H). Collectively, these results establish that FGFR3 inhibition drives CTLs and NK cells recruitment and activation in the UC TME through the STAT5–IRF2–CCL4 chemokine axis, both in vitro and in vivo.

## COMBINATION OF ERDAFITINIB (FGFR3 INHIBITOR) AND NIVOLUMAB (ICI) THERAPY IS SYNERGISTIC IN *FGFR3*–MUTANT UC

Erdafitinib‐induced remodeling of the tumor immune microenvironment in *FGFR3*–mutant UC prompted the evaluation of its combination with ICI (nivolumab).

In UCOs‐PBMCs co‐cultures, the combination further reduced terminally exhausted PD‐1⁺TIM‐3⁺ T cells and achieved superior tumor killing compared to erdafitinib alone (Figure S8). In vivo, in the FGFR3^Y373C^ MB49 syngeneic mouse model (Figure 2O), both monotherapies inhibited tumor growth, but their combination demonstrated markedly superior efficacy with greater suppression of tumor progression and weight (Figure 2P, Figure S9A,B). Immunohistochemistry showed enhanced CD3⁺ and CD8⁺ T cell infiltration upon erdafitinib treatment, further augmented by the combination (Figure S9C–F). Overall, erdafitinib primes the TME for enhanced ICI response, yielding synergistic antitumor effects both in vitro and in vivo.

While this study provides mechanistic insights into immunotherapy resistance in UC, several limitations should be acknowledged. First, while the integration of multi‐omics and functional assays provides a multi‐layered view of *FGFR3*‐driven immune modulation, the number of patient samples still needs to be improved. Second, the syngeneic MB49 model, while useful for immune phenotyping, lacks human tumor–stroma interactions and may not fully mirror *FGFR3*–mutant UC pathophysiology. However, our study integrates multi‐omics profiling, a novel PDOs‐PBMCs co‐culture system, and in vivo models into a comprehensive experimental framework to dissect the mechanisms underlying immunotherapy resistance in UC compared to previous studies.

In conclusion, this study delineates a comprehensive mechanistic framework linking oncogenic FGFR3 signaling to immune evasion and immunotherapy resistance in UC. Specifically, the STAT5–IRF2–CCL4 signaling axis orchestrates interferon responses, antigen presentation, and chemokine‐driven recruitment of immune cells within the TME, thereby shaping antitumor immunity and therapeutic outcomes. This study provides a strong pre‐clinical rationale for the combination therapy of the FGFR3 inhibitor and immunotherapy (Figure 2Q).

## AUTHOR CONTRIBUTIONS

**Shan Jiang**: Conceptualization; investigation; writing—original draft; software; methodology; validation; visualization; writing—review and editing; resources; supervision; data curation; formal analysis; project administration. **Yuxuan Song**: Validation; visualization; writing—review and editing; project administration; formal analysis; software; data curation; supervision; resources. **Yun Peng**: Conceptualization; investigation; writing—original draft; methodology; software; formal analysis; project administration; writing—review and editing; resources; data curation. **Ran Yan**: Conceptualization; investigation; visualization; validation; methodology; formal analysis; project administration. **Yunze Niu**: Conceptualization; investigation; visualization; validation; methodology; software; project administration; formal analysis; resources; data curation. **Baoqiang Chen**: Methodology; validation; software; formal analysis; project administration; resources; supervision; data curation. **Jiaxing Lin**: Methodology; validation; visualization; software; formal analysis; project administration; data curation; supervision. **Jilin Wu**: Methodology; validation; writing—review and editing; visualization; investigation; conceptualization. **Shixiang Wang**: Methodology; data curation. **Yiqing Du**: Conceptualization; investigation; funding acquisition; writing—original draft; project administration; formal analysis; software. **Caipeng Qin**: Conceptualization; investigation; resources; supervision; data curation; formal analysis; software. **Yihan Lin**: Conceptualization; investigation; writing—original draft; methodology; validation; visualization; writing—review and editing; project administration; formal analysis; software; data curation; resources; supervision. **Tao Xu**: Conceptualization; investigation; funding acquisition; writing—review and editing; visualization; validation; methodology; software; formal analysis; project administration; resources; supervision; data curation. All authors have read the final manuscript and approved it for publication.

## CONFLICT OF INTEREST STATEMENT

The authors declare no conflicts of interest.

## ETHICS STATEMENT

All UC tissues were obtained with written informed consent from all patients prior to participation in the study. The Ethical Review Committee of Peking University People's Hospital reviewed and approved this study and the use of UC tissues (2026PHB128‐001). All animal experiments were reviewed and approved by the Ethics Committee for Laboratory Animal Research at Peking University Health Science Center (2025PHB139‐001, 2019PHB133‐01). All aspects of this study complied with the Declaration of Helsinki.

## Supporting information


**Figure S1.** *FGFR3* mutation status, expression profiles, and T cell cytotoxicity and exhaustion scores.
**Figure S2.** Gene expression levels and multicolor immunofluorescence images.
**Figure S3.** Generation of tumor‐reactive T cells via co‐culture system.
**Figure S4.** Flow cytometric and immunofluorescence analysis of T‐cell phenotype, FGFR3 expression, and organoid cytotoxicity in *FGFR3*‐mutant UC models.
**Figure S5.** FGFR3 inhibition increases NK cell proportion/function in co‐culture via IRF2‐dependent IFN‐mediated anti‐tumor immunity.
**Figure S6.** STAT5 inhibition suppresses IFN‐stimulated gene expression, which is partially rescued by combined erdafitinib treatment in co‐culture system.
**Figure S7.** FGFR3 inhibiting promotes chemokine secretion and recruits NK cells into TME via STAT5‐IRF2 axis.
**Figure S8.** Erdafitinib combined with anti‐PD‐1 immunotherapy reduces T‐cell exhaustion and enhances cytotoxic function in co‐culture system.
**Figure S9.** Combination of FGFR3 inhibition (erdafitinib) and anti‐PD1 immunotherapy is synergistic in *FGFR3*‐mutant UC.

**Table S1.** Clinical and molecular characteristics of UC patient cohort.
**Table S2.** Summary of UCOs‐PBMCs co‐culture outcomes across 6 UC patients.
**Table S3.** Homer known Motif enrichment results (homer_*STAT5_IRF2*).
**Table S4.** Differentially expressed genes (DEGs) identified in bulk RNA‐seq analysis.
**Table S5.** Research resource identifiers (RRIDs) for key reagents and resources.

## Data Availability

The data that support the findings of this study are available on request from the corresponding author. The data are not publicly available due to privacy or ethical restrictions. Due to ethical and legal restrictions, de‐identified individual participant data and the accompanying data dictionary cannot be made publicly available. All data are available upon request from the corresponding author, subject to local rules and regulations. The data and scripts used are saved in GitHub (https://github.com/Yunuuuu/UC_Immuno_Erda). Public data used in the present study are available from the R package IMvigor210CoreBiologies (http://research-pub.gene.com/IMvigor210CoreBiologies/). Supplementary materials (methods, figures, tables, graphical abstract, slides, videos, Chinese translated version, and updated materials) can be found in the online DOI or iMeta Science http://www.imeta.science/.
