# Supplementary material for: Patient‐derived organoid‐immune co‐cultures integrated with multi‐omics reveal immunotherapy resistance mechanisms in urothelial carcinoma
Source: Imeta. 2026 May 7;5(3):e70130. doi: 10.1002/imt2.70130 (PMC13377408; doi:10.1002/imt2.70130)
Supplement: Supplementary file 1 — Figure S1. FGFR3 mutation status, expression profiles, and T cell cytotoxicity and exhaustion scores. Figure S2. Gene expression levels and multicolor immunofluorescence images. Figure S3. Generation of tumor‐reactive T cells via co‐culture system. Figure S4. Flow cytometric and immunofluorescence analysis of T‐cell phenotype, FGFR3 expression, and organoid cytotoxicity in FGFR3‐mutant UC models. Figure S5. FGFR3 inhibition increases NK cell proportion/function in co‐culture via IRF2‐dependent IFN‐mediated anti‐tumor immunity. Figure S6. STAT5 inhibition suppresses IFN‐stimulated gene expression, which is partially rescued by combined erdafitinib treatment in co‐culture system. Figure S7. FGFR3 inhibiting promotes chemokine secretion and recruits NK cells into TME via STAT5‐IRF2 axis. Figure S8. Erdafitinib combined with anti‐PD‐1 immunotherapy reduces T‐cell exhaustion and enhances cytotoxic function in co‐culture system. Figure S9. Combination of FGFR3 inhibition (erdafitinib) and anti‐PD1 immunotherapy is synergistic in FGFR3‐mutant UC. [file IMT2-5-e70130-s002.docx]

**Supporting information to**

**Patient-derived organoid-immune co-cultures integrated with multi-omics reveal immunotherapy resistance mechanisms in urothelial carcinoma**

**Running title:** Organoids and multi-omics uncover UC immunotherapy resistance mechanisms

Shan Jiang^1,2#^, Yuxuan Song^1#^, Yun Peng^1#^, Ran Yan^2#^, Yunze Niu^3#^, Baoqiang Chen^2^, Jiaxing Lin^4^, Jilin Wu^1^, Yiqing Du^1^, Caipeng Qin^1^, Yihan Lin^2*^, Tao Xu^1*^

**^1^**Department of Urology, Peking University People's Hospital, Beijing 100044, China

**^2^**Center for Quantitative Biology and Peking-Tsinghua Center for Life Sciences, Academy for Advanced Interdisciplinary Studies, Peking University, Beijing 100871, China

**^3^**Beijing Chaoyang Hospital, Capital Medical University, Beijing 100020, China

**^4^**Department of Urology, Fuzhou University Affiliated Provincial Hospital, Fuzhou 350001, China

**^#^**These authors contributed equally: Shan Jiang, Yuxuan Song, Yun Peng, Ran Yan, Yunze Niu

^*^Correspondence: [xutao@pkuph.edu.cn](mailto:_x0005_xutao@pkuph.edu.cn) (Tao Xu); [yihan.lin@pku.edu.cn](mailto:yihan.lin@pku.edu.cn) (Yihan Lin).

**Supplementary methods**

**Patient‑derived tissue samples**

This study included urothelial carcinoma specimens collected at Peking University People’s Hospital between 2022 and 2025. All samples were surgically resected, pathologically confirmed, and obtained from patients who had not received prior systemic therapy. Fresh tissues designated for single-cell RNA sequencing (scRNA-seq) were preserved in tissue stabilization solution, whereas samples for whole exome sequencing (WES) were immediately stored at -80℃. In total, eight tumor specimens were collected, with detailed usage summarized in Table S1. Written informed consent was obtained from all participants prior to inclusion. The study was approved by the Ethics Committee of Peking University People’s Hospital (ID: 2026PHB128-001), and all procedures were conducted in accordance with the ethical standards of the Institutional Review Board.

**Preparation of single-cell suspensions**

Tumor specimens were transported in MACS Tissue Storage Solution (Miltenyi Biotec, cat#130-100-008) at 4℃ immediately after surgical resection. Primary tumor tissues were rinsed with phosphate-buffered saline (PBS; Thermo Fisher Scientific), minced into~1mm^3^ fragments, and homogenized using a UTTD disperser (ULTRA-TURRAX® Tube Drive; IKA, Germany). The tissue suspension was first digested with 0.25% trypsin (Gibco, Life Technologies), quenched with H1640 medium supplemented with 10% fetal bovine serum (FBS; Gibco, Life Technologies), and subsequently transferred to digestion medium containing collagenase IV (100 U/ml; Gibco, Life Technologies) and dispase (0.6 U/ml; Gibco, Life Technologies). Following enzymatic digestion, the samples were passed through a 70-μm nylon mesh filter, centrifuged, and resuspended in ice-cold red blood cell lysis buffer (Solarbio). The resulting cell suspension was then filtered through a 40-μm nylon mesh. Finally, the cell pellet was resuspended in 1 ml of Dulbecco’s PBS (Solarbio), and live and clumped cells were quantified using an automated cell counter (Countstar).

**Droplet-based single-cell sequencing**

scRNA sequencing libraries were prepared using the Single Cell 3′ Library and Gel Bead Kit V3 (10X Genomics) and the Chromium Single Cell A Chip Kit (10X Genomics). Cell suspensions were loaded onto the Chromium Single Cell Controller (10X Genomics) to generate single-cell gel beads in emulsion (GEMs) following the manufacturer’s protocol. Briefly, single cells were suspended in PBS containing 0.04% bovine serum albumin, with approximately 10,000 cells loaded per channel. Captured cells were lysed, and released RNA was barcoded via reverse transcription within individual GEMs. Reverse transcription was performed at 53℃ for 45 min, followed by 85℃ for 5 min, and samples were subsequently held at 4℃. Complementary DNA (cDNA) was generated and amplified, and quality was assessed using an Agilent 4200 instrument according to the manufacturer’s instructions. scRNA-seq libraries were then constructed using the 10X Genomics Single Cell 3′

Library Gel Bead Kit V3, and sequencing was carried out on an Illumina HiSeq PE150 platform.

**Single-cell RNA sequencing data processing**

Raw gene expression matrices for each sample were generated using the Cell Ranger pipeline (v7.1.0) with the human reference genome GRCh38. Filtered matrices were analyzed using the Seurat R package (v4.0.0) [1] or Bioconductor single cell toolkits [2]. Doublets were computationally identified and removed on a per-sample basis using DoubletFinder (v2.0) with default parameters. Low-quality cells were excluded after filtering detected gene numbers, unique molecular identifiers (UMIs), mitochondrial gene content and ribosomal gene content. Specifically, for the *FGFR3*–mutant patient-derived tissue samples, the number of cells passing QC were 6,218, 12,272, 13,020, and 15,261, while for the *FGFR3*–wild patient-derived tissue samples, the number of cells passing QC were 7,054, 8,629, 8,999, and 13,081. The filtered expression matrix was normalized, and the 2,000 most highly variable genes were selected to capture cell-to-cell transcriptional heterogeneity. Principal component analysis (PCA) was performed using these variable genes for dimensionality reduction. To integrate multiple samples, batch effects were corrected using the Harmony (v1.2.1) [3] or fastMNN algorithm (v0.1.1) [4]. Cells were then subjected to unsupervised clustering, and results were visualized using uniform manifold approximation and projection (UMAP).

**Cell type annotation and cluster marker identification**

Following nonlinear dimensionality reduction, cells were projected into two-dimensional space using UMAP. Cells with shared transcriptional features formed distinct clusters, which were annotated based on the expression of canonical markers for specific cell types. Clusters co-expressing markers from two or more cell types were designated as doublets, while clusters lacking expression of canonical markers were considered low-quality. Both doublet and low-quality clusters were excluded from downstream analyses.

**Subclustering of major cell types**

For each major cell type, cells were first extracted from the integrated dataset. These subsets were then re-integrated to perform finer subclustering. Following integration, gene expression values were scaled to unit variance. Subsequent scaling, principal component analysis (PCA), and clustering were performed as described above.

**Gene set enrichment analysis**

Pathway analyses were conducted using the Hallmark (H) and Gene Ontology (C5) gene sets from the Molecular Signatures Database [5]. Gene set enrichment analysis (GSEA) was applied to estimate pathway activity across different groups, using the fgsea R package (v1.24.0) with default parameters.

**Ro/e Analysis for cell type proportion comparison between groups**

To assess differences in cell type composition between groups, we applied the ratio of observed to expected cell (Ro/e) analysis [6]. Contingency tables were constructed for each group, categorizing cells by cluster. For each combination of cluster and group, expected cell counts were calculated using a chi-squared test-based approach to evaluate deviations from random distribution. Ro/e values were then compared between groups, with values >1 indicating enrichment and values < 1 indicating depletion of specific clusters. This analysis enabled identification of group-specific cell type preferences, providing insights into biological variability between conditions.

**Bulk RNA sequencing data analysis**

Raw sequencing data quality was first evaluated using FastQC (v0.12.0) to ensure high-quality reads. Cleaned reads were aligned to the GRCh38 reference genome. The HISAT2 aligner (v2.2.1) was used to construct the reference genome index and perform splice-aware alignment of paired-end reads, incorporating gene model annotation files to improve alignment accuracy. Gene-level read counts were then generated using featureCounts (v2.0.6).

Differential gene expression analysis was conducted using the DESeq2 R package (v1.38.3) to identify genes significantly up- or downregulated under different conditions. Genes were considered significantly differentially expressed if they met the thresholds of adjusted *p*-value (*p* adj) < 0.05 and |log₂ (fold change) | > 1. Gene Ontology enrichment analysis of differentially expressed genes was performed using the clusterProfiler R package (v4.15.0).

**Whole-exome sequencing data analysis**

Tumor DNA from FFPE samples was extracted using the Maxwell 16 FFPE Plus LEV DNA Purification Kit (Promega, AS1135), and genomic DNA from peripheral blood was purified using the OMEGA Blood DNA Kit, following the manufacturers’ protocols. DNA was sheared to an appropriate fragment size for library construction, and libraries were prepared using the Agilent SureSelect Human All Exon V6 Kit. For FFPE samples with total DNA < 200 ng, libraries were prepared using the KAPA Hyper Prep Kit. Targeted DNA fragments were captured and sequenced on the Illumina NovaSeq 6,000 platform, and raw base calls and fluorescence intensities were processed into FASTQ files. Somatic mutations were called based on the GATK best practices workflow [7,8]. Copy number analysis was performed using alleleCount and ASCAT (v3.1.2) [9].

**Public dataset data analysis**

Bulk RNA-seq expression data and clinical data of IMvigor210 cohort were extracted from the IMvigor210CoreBiologies R package (version 1.0.0). The *FGFR3* mutation status was determined based on the gene mutation profile obtained through whole exome sequencing. We included all patients with *FGFR3* mutations.

**Real‑time PCR**

Total RNA was extracted using TRIzol™ Reagent (Thermo Fisher), and 1 µg of RNA was reverse-transcribed using the High-Capacity cDNA Reverse Transcription Kit (TransScript, Cat# AT341) according to the manufacturer’s instructions. Quantitative real-time PCR (qRT-PCR) was performed using SYBR Green Master Mix with gene-specific primers. Relative mRNA expression levels were normalized to the endogenous control GAPDH and calculated using the comparative Ct method (2^−ΔΔCt^).

**Organoid culture**

Tumor samples were washed twice with cold PBS and minced into small fragments. Two portions of minced tissue were snap-frozen and stored at −80℃ for DNA and RNA extraction, while the remaining tissue was used for cancer cell isolation. For organoid derivation, tissues were further minced in a 10-cm culture plate and dissociated in AdDMEM/F12 containing 1× Collagenase/hyaluronidase and ROCK inhibitor Y-27632 dihydrochloride (1 µM) for 1 h at 37℃. Digested tissue was centrifuged at 400 × g for 4 min, washed once with DPBS, and incubated in 5 mL TrypLE Express supplemented with Y-27632 (1 µM) for 8 min at 37℃. Digestion was quenched with 10 mL AdDMEM/F12 supplemented with 1% penicillin/streptomycin, 1% Glutamax, 1% HEPES, and 20% FBS. The pellet was resuspended in 2 mL AdDMEM/F12 with 20% FBS and mechanically dissociated by pipetting. Cells were filtered through a 100-µm strainer to remove undigested clusters, centrifuged, and resuspended in cold organoid medium mixed with cold Matrigel. Approximately 100,000 cells in 100 µL Matrigel droplets were plated per well of a 12-well plate. Plates were inverted to solidify Matrigel for 10 min at 37℃ with 5% CO_2_, after which 2 mL of UC organoid medium was added to each well. Medium was refreshed every 2–3 days.

**Isolation of human peripheral blood mononuclear cells (PBMCs) from blood**

PBMCs were isolated as follows. Blood was diluted 1:1 with 0.1% BSA in PBS (PBSA) filtered through a 0.2-µm membrane. The diluted blood was carefully layered over 15 mL Lympholyte H in a 50-mL tube by tilting the tube horizontally to preserve layer separation. Tubes were centrifuged at 1,200 × g for 15 min at room temperature without brake or acceleration. Following centrifugation, the PBMC layer, located between the plasma and red blood cells, was carefully collected and transferred to a new tube. Cells were washed twice with 0.1% PBSA (1,000 × g, 7 min, room temperature) and once at 300 × g for 7 min to remove platelets. Residual erythrocytes were eliminated by incubating the cells in RBC lysis buffer for 5 min at 37℃, followed by a final wash at 300 × g for 7 min. PBMCs were counted using a hemocytometer or automated cell counter and cryopreserved for subsequent experiments.

**Construction of co-culture system**

For co-culture, 96-well U-bottom plates were pre-coated with anti-CD28 antibody (5 µg/mL) and incubated overnight at 4℃. Thawed PBMCs were treated with benzonase and cultured overnight in IL-2–supplemented medium (150 U/mL) at 2 × 10⁶ cells/mL. Tumor organoids were dissociated into single cells using TrypLE, washed, and resuspended at 1 × 10⁵ cells/mL. PBMCs were resuspended in medium containing 300 U/mL IL-2 at 1 × 10⁶ cells/mL. PBMCs and tumor cells were combined at a 10:1 ratio, and 200 µL of the mixture was seeded per well into the anti-CD28–coated plate. Cultures were maintained at 37℃, with medium refreshed or cultures split every 2–3 days. On day 7, PBMCs were collected without separating tumor cells and re-cultured with fresh organoids. By day 14, T cells constituted approximately 90% of the PBMC population, ready for downstream assays.

**Cell line culture**

The murine bladder cancer cell line MB49 (CTCC, Cat#SCC148, RRID: CVCL_7076) was obtained from the Meisen Cell Technology (CTCC). MB49 cells were maintained in Dulbecco’s Modified Eagle Medium (DMEM, Gibco) supplemented with 10% fetal bovine serum (FBS, Cytiva) and 1% penicillin-streptomycin (Gibco). Cells were used within 5 passages after thawing and cultured in an incubator with 5% CO_2_ at 37 °C.

Cell line authentication was performed by Meisen Cell Technology (Suzhou, China) using short tandem repeat (STR) profiling at 18 loci plus one human-specific marker (TH01). The submitted profile showed a 95.24% match to the ExPASy reference database profile for MB49, confirming its identity with no evidence of misidentification or cross-contamination. The original STR report is provided in Supplementary File S1.

Mycoplasma contamination was tested using the PCR Mycoplasma Detection Kit (HUABIO, Cat# K0103) following the manufacturer’s instructions. MB49 cells culture supernatant (collected after 72 hours of culture) was analyzed and no mycoplasma-specific bands were detected, confirming the absence of mycoplasma contamination. The original PCR gel image and report are provided in Supplementary File S2.

**Lentivirus generation and gene transfer**

Human or mouse wild-type (WT) c*FGFR3* cDNA was cloned into the pHR-SFFV-KRAB-dCas9-P2A-mCherry vector and used as a template for site-directed mutagenesis. The activating Y373C mutation was introduced by PCR using mutation-specific primers and verified by Sanger sequencing. Lentiviral vectors encoding either mutant c*FGFR3* or an empty control were packaged in HEK293T cells. Viral supernatants were collected and used to transduce MB49 cells in the presence of polybrene (IGE Biotechnology). Successful transduction was confirmed by mCherry fluorescence and Western blotting.

**Flow cytometry**

Cells were harvested and washed with DPBS or staining buffer. Surface staining was performed by incubating cells with fluorochrome-conjugated antibodies for 20–30 min at 4℃ in the dark, followed by two washes. Cells were fixed with 4% paraformaldehyde for 15–20 min at room temperature and then washed. For intracellular staining, cells were permeabilized using a suitable buffer and incubated briefly, followed by incubation with antibodies diluted in permeabilization buffer for 30–60 min at 4℃ in the dark. After washing, cells were resuspended in staining or FACS buffer and analyzed using a flow cytometer.

**Western blot**

Cells were lysed on ice in RIPA buffer (Beyotime, P0013B) supplemented with protease and phosphatase inhibitors (Sigma-Aldrich) for 20 min. The lysates were centrifuged at 12,000 × g for 15 min at 4℃, and protein concentrations were quantified using a BCA protein assay kit (Sigma-Aldrich). Equal amounts of protein were separated by SDS-PAGE and transferred onto nitrocellulose membranes. After blocking with 5% non-fat dry milk in TBS containing 0.1% Tween-20 (TBST) for 1 h at room temperature, membranes were incubated overnight at 4℃ with primary antibodies. Following washes in TBST, membranes were incubated with HRP-conjugated secondary antibodies (goat anti-mouse or goat anti-rabbit IgG; Abcam) and visualized using a Tanon chemiluminescence imaging system.

**Inguinal tumor model**

*FGFR3^Y373C^* MB49 cells (1 × 10⁶) were subcutaneously inoculated into the inguinal region of mice. Tumor size was measured every 2–3 days using an electronic caliper, and volumes were calculated as 0.5 × L × W². When tumors reached a predefined size, mice were randomized into treatment groups. Erdafitinib was formulated in 20% hydroxypropyl-β-cyclodextrin (HP-β-CD) and administered orally at 25 mg/kg twice daily. Anti-PD-1 antibodies were delivered by intraperitoneal injection at 10 mg/kg every 3 days. At the study endpoint, mice were sacrificed, and tumors were excised for downstream analyses. In mice experiments, the anti-mouse PD-1 antibody (catalog no. P362) from Leinco Technologies was used.

**CUT&Tag**

CUT&Tag assays were performed using the Hyperactive Universal CUT&Tag Assay Kit for Illumina (Vazyme, TD903) according to the manufacturer’s protocol. Briefly, 5 × 10⁵ urothelial carcinoma (UC) cells were harvested, washed once with 500 µL wash buffer, and bound to ConA beads for 10 min at 25℃. Cells were then incubated overnight at 4℃ with 1 µg of the target-specific antibody (anti-STAT5 or anti-IRF2) or 1 µg of normal IgG (as negative control) (Specific catalog numbers are listed in Table S5). After three washes with DIG wash buffer, cells were incubated with 0.04 µM pA/G-Tn5 transposase for 1 h at 25℃, followed by three washes with DIG 300 buffer. Tagmentation was carried out by resuspending the cells in tagmentation buffer and incubating at 37℃ for 1 h. The reaction was terminated by adding proteinase K, buffer LB, and DNA extraction beads, followed by incubation at 55℃ for 10 min. Cells were then separated using a magnetic stand, washed twice with 80% ethanol, and DNA was eluted in nuclease-free water. Sequencing libraries were generated using the TD903 Hyperactive Universal CUT&Tag Assay Kit in combination with the TruePrep Index Kit V2 for Illumina (Vazyme, TD202).

**CUT&Tag data processing**

Raw paired-end reads were trimmed for adaptor sequences using Trim Galore (v0.6.6). Cleaned reads were aligned to the mm10 reference genome using Bowtie 2 (v2.4.2). Duplicates were marked and removed using Picard, and SAM files were converted and sorted into BAM format with Samtools (v1.11). Peaks were called with MACS2 (v2.2.7.1). Signal normalization from sorted BAM files was performed using bamCoverage in deepTools, and computeMatrix was used to calculate signal distribution around MACS2-called peak centers using the “reference-point” model. Signal profiles were visualized with plotProfile in deepTools.

**Statistical analysis**

Correlation analyses were performed using the Spearman method to assess the strength and direction of associations. For continuous variables, the Wilcoxon test or Student t-test was used for statistical analysis. For categorical variables, the Chi-square test was used for statistical analysis. Statistical analyses were conducted using R (v4.1.2) and GraphPad Prism (v10.0). Results with *p* < 0.05 were considered statistically significant. Error bars in figures represent the standard deviation (SD) from independent experiments.

For comparisons involving three or more groups, appropriate statistical methods were employed. Data conforming to a normal distribution were first analyzed using one-way analysis of variance (ANOVA) to assess overall differences between groups. Subsequently, pairwise comparisons among three or more groups were conducted with post hoc multiple comparison corrections using the Benjamini-Hochberg procedure to control the false discovery rate. These analyses were based on Student's t-test. All reported *p*-values are adjusted to minimize the risk of false positives arising from multiple comparisons

The two-sided *p* < 0.05 and adjusted *p* < 0.05 were set as statistical significance.

For high-dimensional data, multiple testing was corrected using the Benjamini-Hochberg false discovery rate (FDR) approach with significance defined as adjusted *p* < 0.05.

**References**

1. Butler, Andrew, Paul Hoffman, Peter Smibert, Efthymia Papalexi, Rahul Satija. 2018. “Integrating single-cell transcriptomic data across different conditions, technologies, and species.” *Nature Biotechnology* 36: 411−420. <https://doi.org/10.1038/nbt.4096>

2. Amezquita, Robert A, Aaron T L Lun, Etienne Becht, Vince J Carey, Lindsay N Carpp, Ludwig Geistlinger, Federico Marini, et al. 2020. “Orchestrating single-cell analysis with Bioconductor.” *Nature Methods* 17: 137-145. https://doi.org/10.1038/s41592-019-0654-x

3. Korsunsky, Ilya, Nghia Millard, Jean Fan, Kamil Slowikowski, Fan Zhang, Kevin Wei, Yuriy Baglaenko, Michael Brenner, Po-Ru Loh, Soumya Raychaudhuri. 2019. “Fast, sensitive and accurate integration of single-cell data with Harmony.” *Nature Methods* 16: 1289−1296. <https://doi.org/10.1038/s41592-019-0619-0>

4. Haghverdi, Laleh, Aaron T L Lun, Michael D Morgan, John C Marioni. 2018. “Batch effects in single-cell RNA-sequencing data are corrected by matching mutual nearest neighbors.” *Nature Biotechnology* 36: 421−427. <https://doi.org/10.1038/nbt.4091>

5. Subramanian, Aravind, Pablo Tamayo, Vamsi K Mootha, Sayan Mukherjee, Benjamin L Ebert, Michael A Gillette, Amanda Paulovich, et al. 2005. “Gene set enrichment analysis: a knowledge-based approach for interpreting genome-wide expression profiles.” *Proceedings of the National Academy of Sciences of the United States of America* 102: 15545−15550. <https://doi.org/10.1073/pnas.0506580102>

6. Zhang, Lei, Xin Yu, Liangtao Zheng, Yuanyuan Zhang, Yansen Li, Qiao Fang, Ranran Gao, et al. 2018. “Lineage tracking reveals dynamic relationships of T cells in colorectal cancer.” *Nature* 564: 268−272. <https://doi.org/10.1038/s41586-018-0694-x>

7. DePristo, Mark A, Eric Banks, Ryan Poplin, Kiran V Garimella, Jared R Maguire, Christopher Hartl, Anthony A Philippakis, et al. 2011. “A framework for variation discovery and genotyping using next-generation DNA sequencing data.” *Nature Genetics* 43: 491−498. <https://doi.org/10.1038/ng.806>

8. McKenna, Aaron, Matthew Hanna, Eric Banks, Andrey Sivachenko, Kristian Cibulskis, Andrew Kernytsky, Kiran Garimella, et al. 2010. “The Genome Analysis Toolkit: a MapReduce framework for analyzing next-generation DNA sequencing data.” *Genome Research* 20: 1297−1303. <https://doi.org/10.1101/gr.107524.110>

9. Peter, Van Loo, Silje H Nordgard, Ole Christian Lingjærde, Hege G Russnes, Inga H Rye, Wei Sun, Victor J Weigman, et al. 2010. “Allele-specific copy number analysis of tumors.” *Proceedings of the National Academy of Sciences of the United States of America* 107: 16910−16915. <https://doi.org/10.1073/pnas.1009843107>

**Supplementary figures**


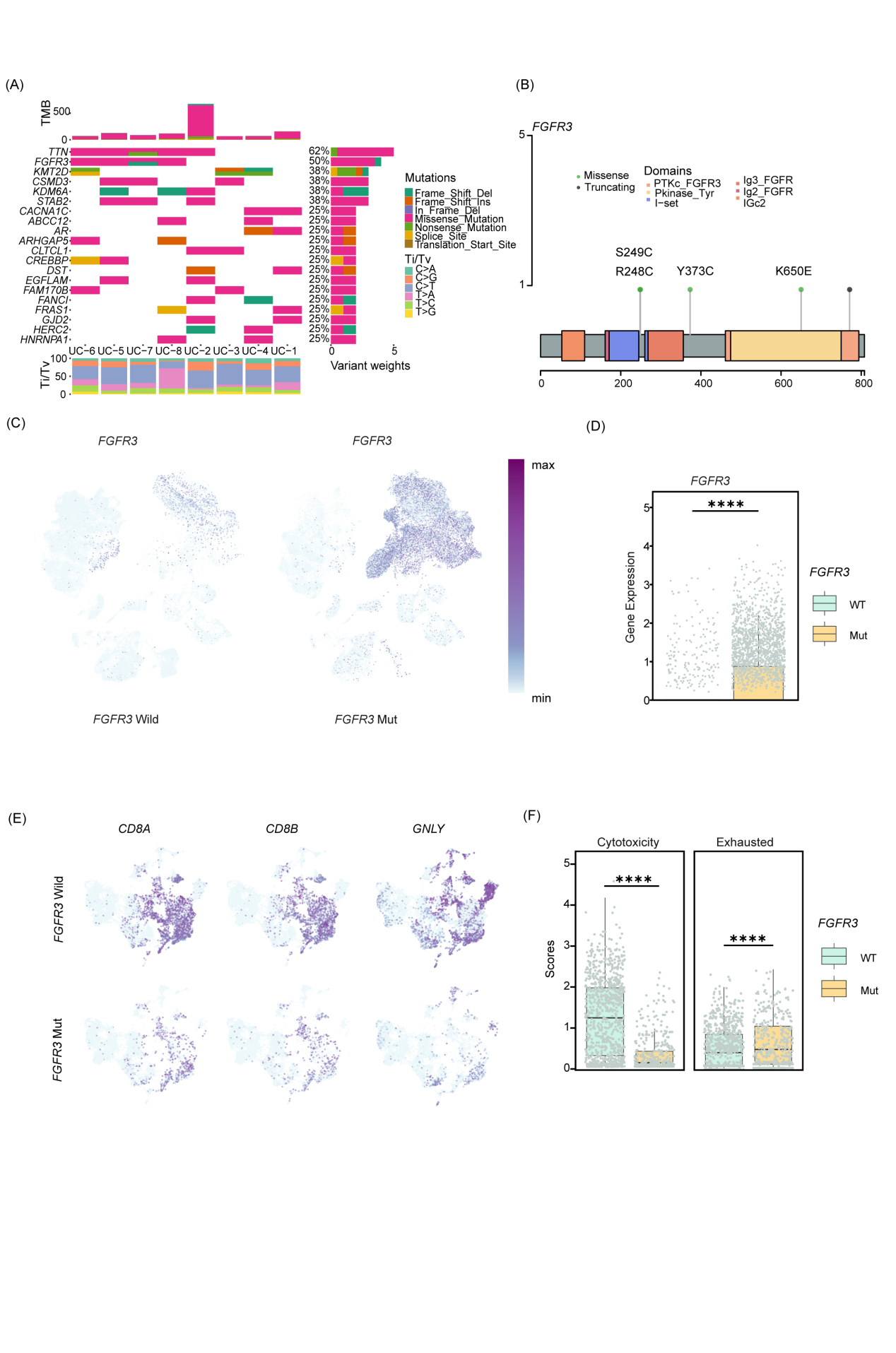


**Figure S1 *FGFR3* mutation status, expression profiles, and T cell cytotoxicity and exhaustion scores. (A)** The oncoplot displays the top 20 genes with the highest mutation frequencies across the samples, highlighting the mutation types and their distribution to provide an overview of key genetic alterations. (B) Lollipop plots illustrating the specific amino acid changes in the *FGFR3* protein. (C) Uniform manifold approximation and projection (UMAP) visualization of all cells colored by expression levels of *FGFR3*. (D) Boxplot comparing the expression levels of *FGFR3* across the indicated groups. (E) UMAP visualization of T cells colored by expression levels of *CD8A*, *CD8B* and *GNLY*. (F) Boxplots showing the distribution of cytotoxicity (left) and exhaustion (right) scores in T cells stratified by *FGFR3* mutation status. Label means **p* < 0.05, ***p* < 0.01, ****p* < 0.001, *****p* < 0.0001. WT: wildtype, Mut: mutant.

**
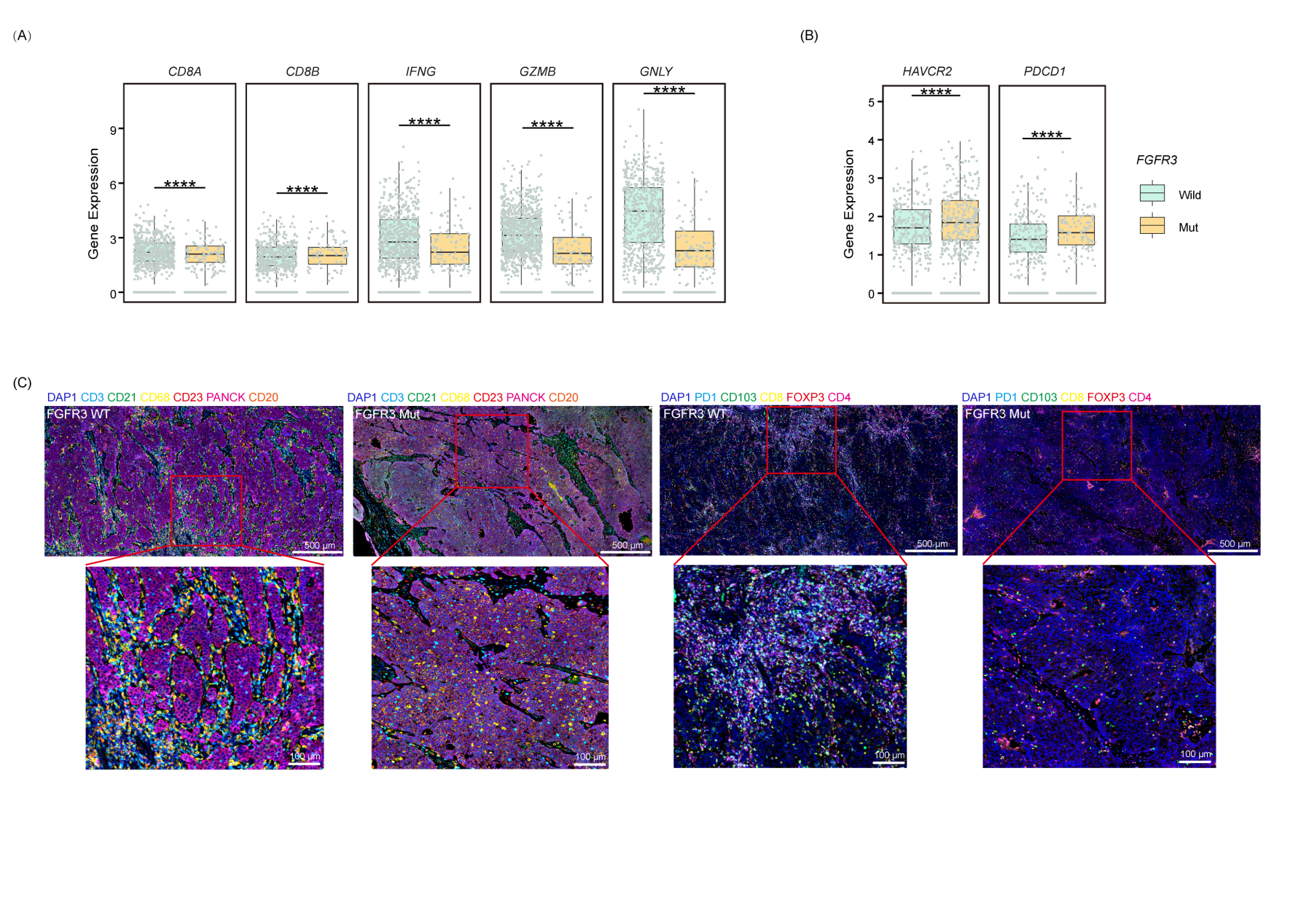
**

**Figure S2 Gene expression levels and multicolor immunofluorescence images.** **(A and B)** Boxplot comparing the expression levels of *CD8A*, *CD8B*, *IFNG*, *GZMB,* and *GNLY* (A), and *HAVCR2* and *PDCD1* (B) across the indicated groups. (C) Multicolor immunofluorescence images of tumor tissue stained for CD3, CD21, CD68, CD23, PANCK, CD20, PD1, CD103, CD8, FOXP3 and CD4 in *FGFR3*–WT and *FGFR3*–mutant UC samples. Nuclei were stained with DAPI (blue). **Scale bar: 500 μm (upper), 100 μm (lower).** Label means **p* < 0.05, ***p* < 0.01, ****p* < 0.001, *****p* < 0.0001. WT: wildtype, Mut: mutant.

**
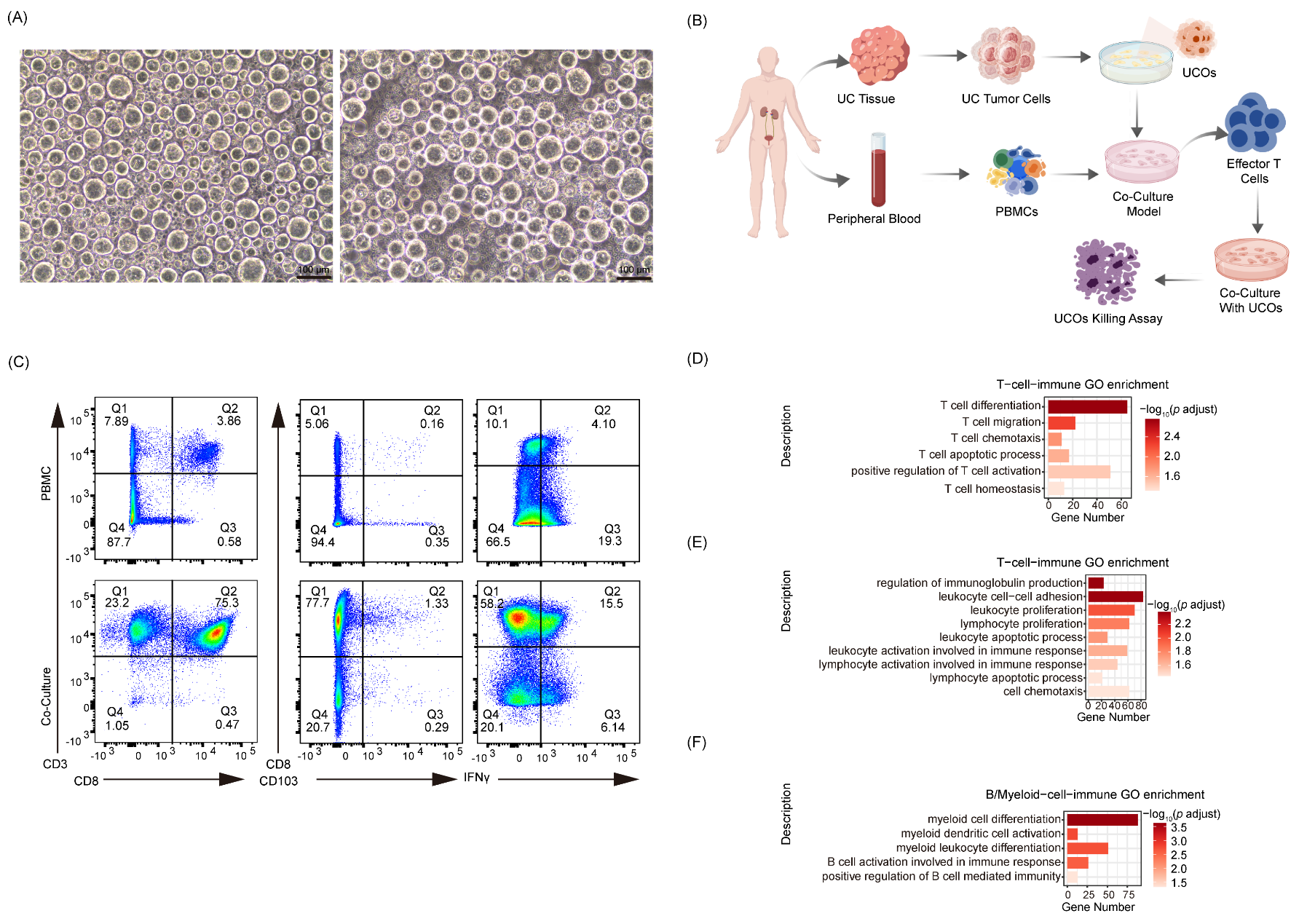
**

**Figure S3 Generation of tumor-reactive T cells via co-culture system. (A)** Bright–field images of urothelial carcinoma organoids (UCOs) during 3D culture. **Scale bar: 100 μm.** (B) Schematic overview of the experimental workflow for co-culture system. Created with BioGDP.com. (C) Flow cytometry analysis of CD3^+^CD8^+^, CD8^+^CD103^+^, and CD8^+^IFNγ^+^ T cells in peripheral blood mononuclear cells (PBMCs) post 14-day co-culturing with autologous UCOs. (D–F) Bar plot summarizing the GO enrichment analysis results of bulk RNA-seq for UCOs-PBMCs, with each bar representing an enriched GO term. Up-regulated genes in UCOs-PBMCs were enriched in immune-related terms.


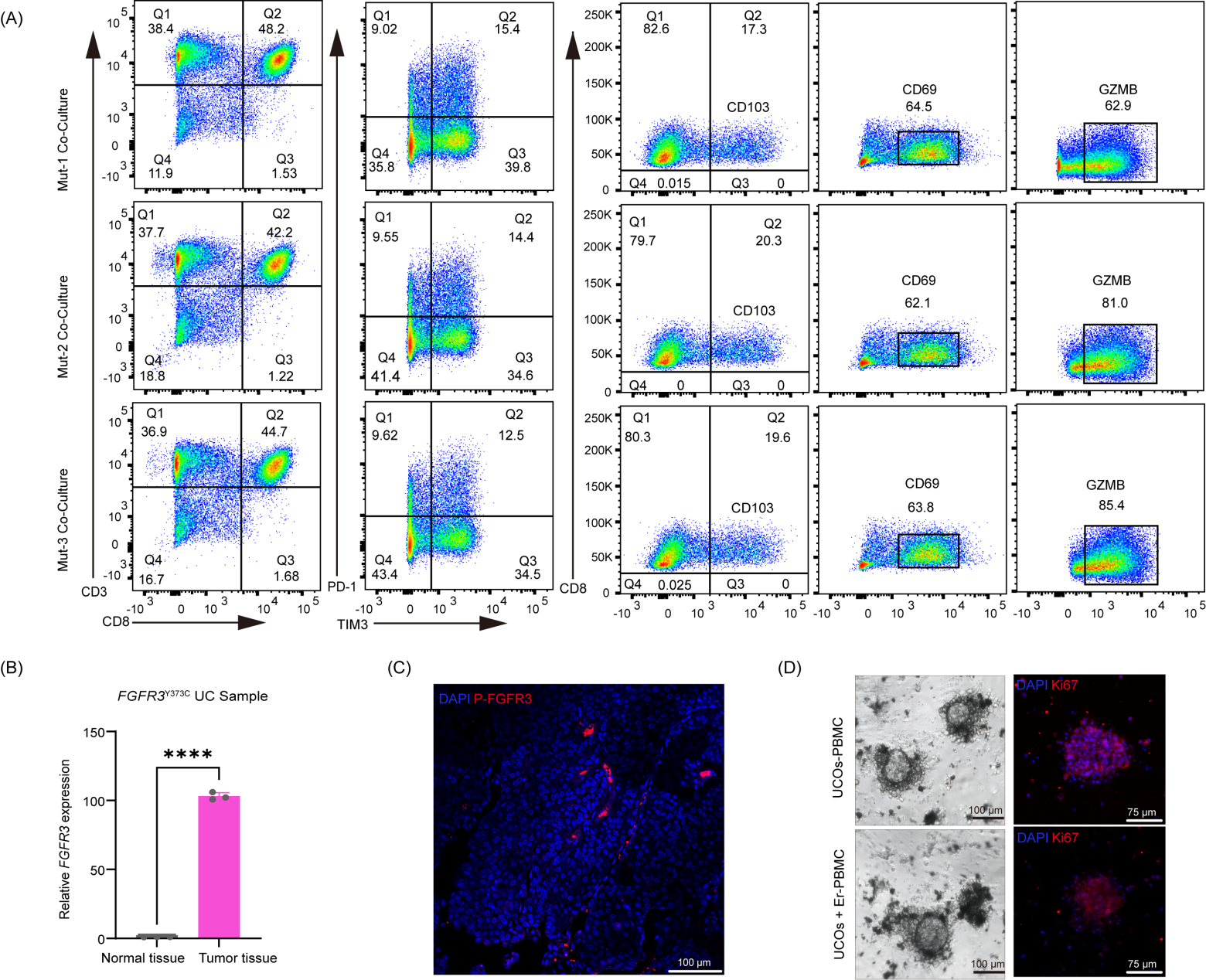


**Figure S4 Flow cytometric and immunofluorescence analysis of T-cell phenotype, FGFR3 expression, and organoid cytotoxicity in *FGFR3*–mutant urothelial carcinoma (UC) models.** (A) Flow cytometry analysis of CD3^+^CD8^+^, PD-1^+^TIM-3^+^, CD8^+^CD103^+^, CD8^+^CD69^+^, and CD8^+^GZMB^+^ T cells in PBMCs post 14-day co-culturing with autologous UCOs derived from 3 *FGFR3*–mutant UC lines. (B) Real-time qPCR validation of *FGFR3* expression in *FGFR3*–mutant UC and adjacent normal tissues. **(C) Immunofluorescence images of *FGFR3***–**wild UC sample stained for phosphorylation of FGFR3 (P-FGFR3), Nuclei were** counterstained **with DAPI (blue). Scale bar: 100 μm.** (D) **Immunofluorescence and bright-field images of UCOs stained for Ki67 in the different co-culture groups, Nuclei were** counterstained **with DAPI (blue). Scale bar: 100 μm** (left), 75 **μm** (right). Label means **p* < 0.05, ***p* < 0.01, ****p* < 0.001, *****p* < 0.0001. Mut: Mutant.

**
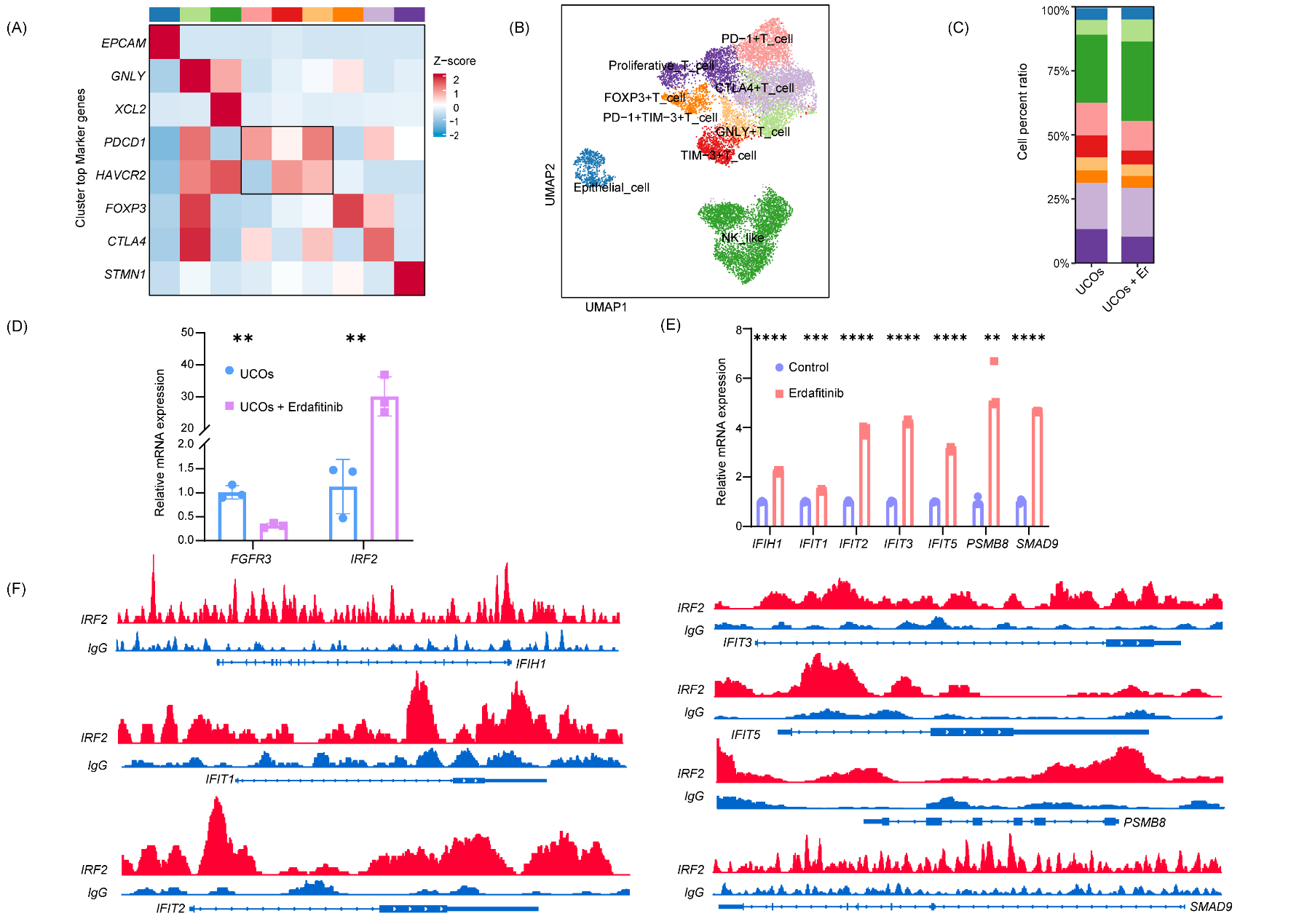
**

**Figure S5 FGFR3 inhibition increases natural killer (NK) cell proportion/function in co-culture via IRF2-dependent IFN-mediated anti-tumor immunity. (A)** Heatmap illustrating the expression levels of cell type–specific marker genes across different cell subtype populations. (B) Uniform manifold approximation and projection (UMAP) plot showing the subtypes. (C) Percentage stacked bar chart depicting the proportions of cellular subpopulations across different UCOs-PBMCs co-culture groups. (D) Real-time qPCR validation of *FGFR3* and *IRF2* in erdafitinib treated UCOs compared with control. (E) CUT&Tag in *FGFR3*–mutant UC cells revealed binding peaks for *IRF2* on the promoters of IFN response genes. (F) Real-time qPCR validation of representative IFN response genes in erdafitinib treated UCOs compared with control. Label means **p* < 0.05, ***p* < 0.01, ****p* < 0.001, *****p* < 0.0001. Er: Erdafitinib.


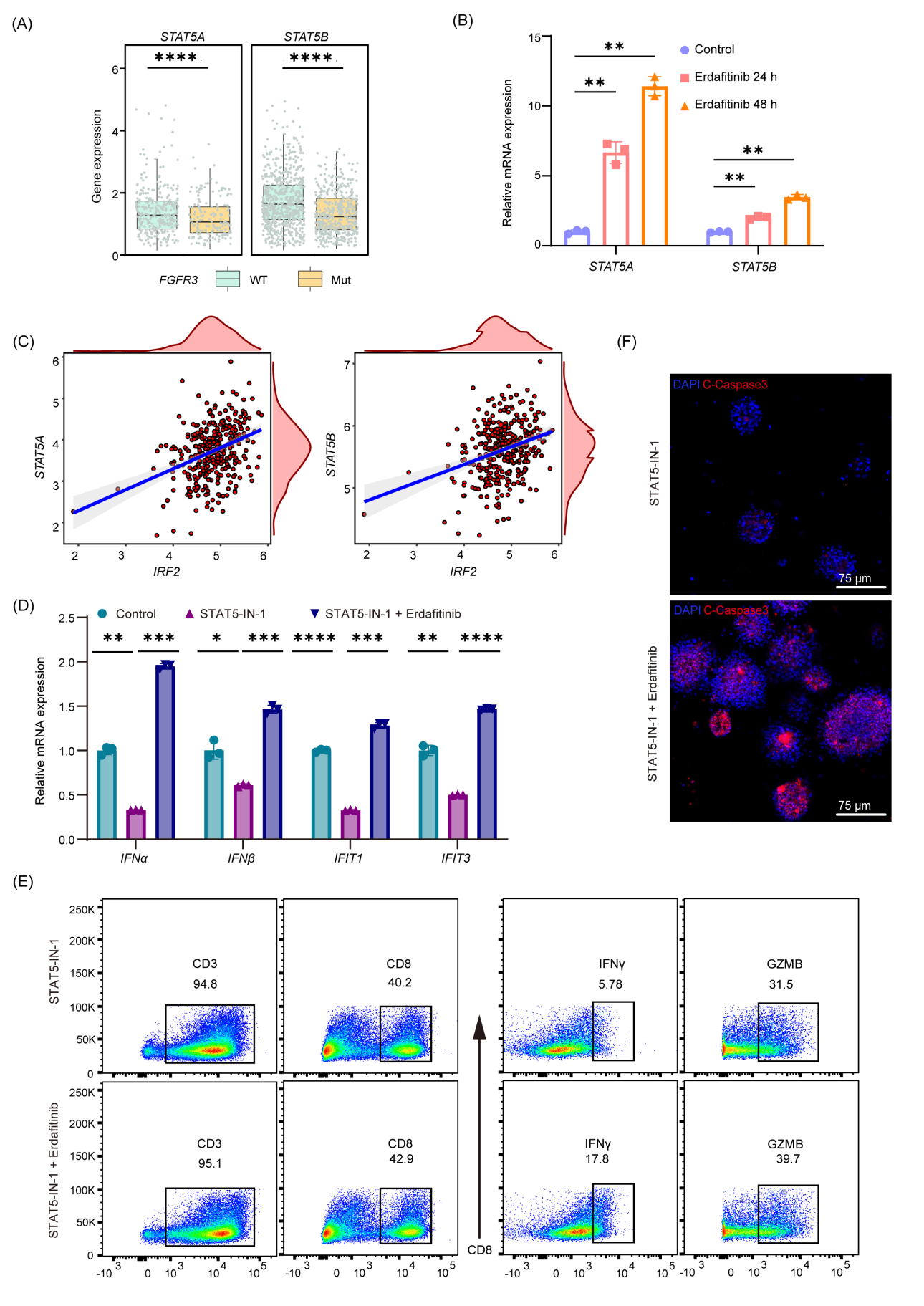


**Figure S6 STAT5 inhibition suppresses IFN-stimulated gene expression, which is partially rescued by combined erdafitinib treatment in co-culture system.** (A) Boxplot comparing the expression levels of *STAT5A* and *STAT5B* between the indicated groups. (B) Real-time qPCR validation of *STAT5A* and *STAT5B* in the indicated groups. (C) Gene expression correlation between *IRF2* vs *STAT5A* and *STAT5B* transcripts in IMvigor 210 immunotherapy trial. (D) Real-time qPCR validation of representative IFN response genes in different groups. (E) Flow cytometric analysis of CD3⁺, CD8⁺, CD8⁺IFN-γ⁺, and CD8⁺GZMB⁺ T cells in PBMCs after 14-day co-culture with autologous UCOs, comparing STAT5-IN-1 treatment alone versus combination with erdafitinib. (F) Immunofluorescence images of UCOs stained for Cleaved-caspase3 (C-caspase3). Nuclei were counterstained with DAPI (blue). **Scale bar: 250 μm**. Label means **p* < 0.05, ***p* < 0.01, ****p* < 0.001, *****p* < 0.0001. WT: wild type, Mut: mutant.


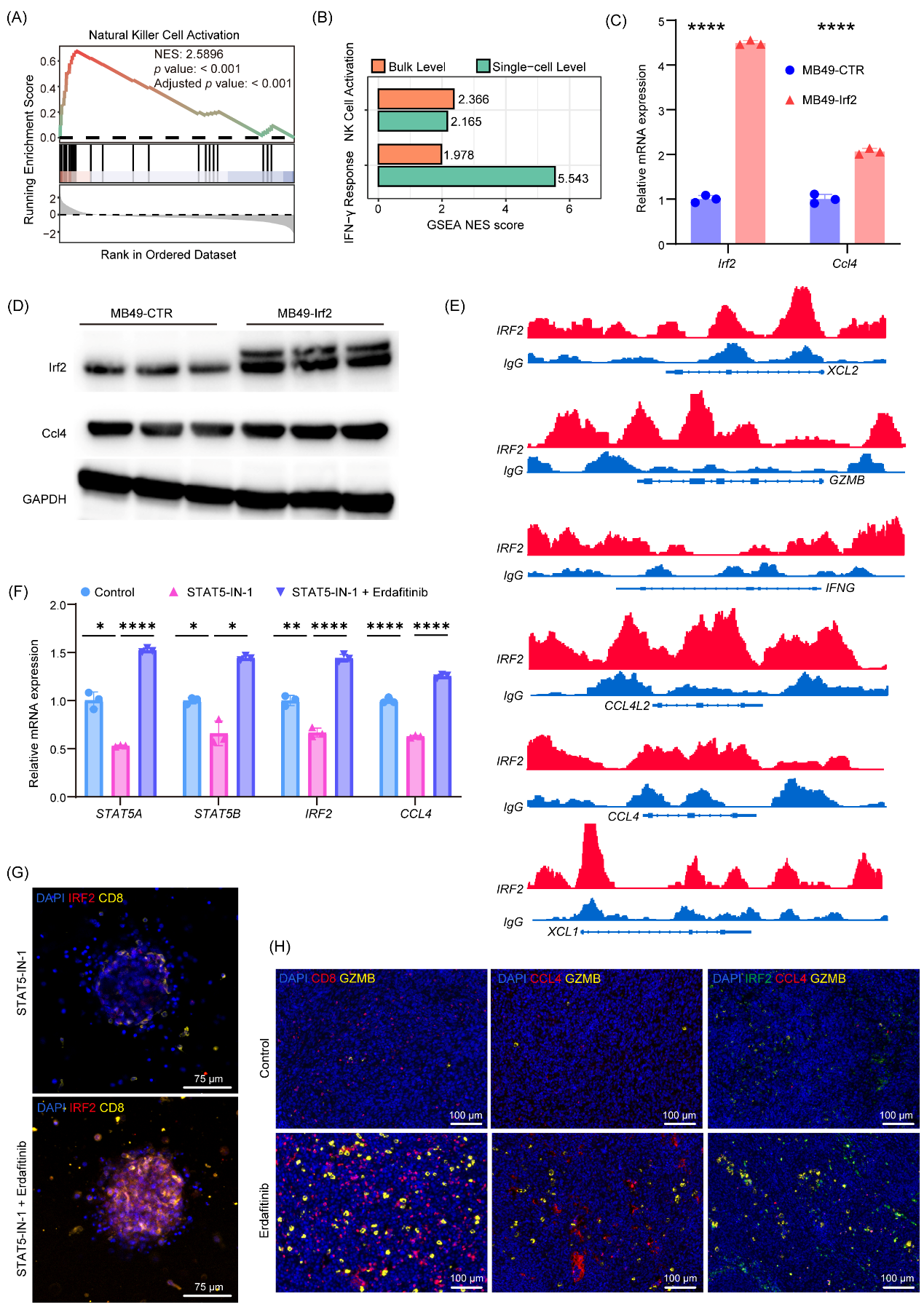


**Figure S7 FGFR3 inhibiting promotes chemokine secretion and recruits NK cells into TME via STAT5*-*IRF2 axis. (A)** GSEA enrichment curve illustrating Natural Killer Cell Activation and T Cell Activation pathways were up-regulated in UCOs-PBMCs co-culture system. (B) GSEA normalized enrichment scores (NESs) of IFNγ response and NK cell activation at the bulk (UCOs–PBMCs versus PBMCs) and single-cell (UCOs-PBMCs versus UCOs+Er-PBMCs) levels. (C) Real-time qPCR validation of Irf2 and Ccl4 in the indicated groups. (D) Western blot analysis of Irf2 and Ccl4 expression in the indicated groups. (E) CUT&Tag in *FGFR3*–mutant UC cells revealed binding peaks for *IRF2* on the promoters of *CCL4L2*, *XCL2*, *CCL4*, *GZMB*, *XCL1*, and *IFNG*. (F) Real-time qPCR validation of *STAT5A*, *STAT5B*, *IRF2*, and *CCL4* in the indicated groups. (G) Immunofluorescence images of UCOs stained for IRF2 and CD8. Nuclei were counterstained with DAPI (blue). **Scale bar: 75 μm**. (H) Immunofluorescence images of tumor tissue stained for CD8, GZMB, CCL4, and IRF2 in *FGFR3*^Y373C^ MB49 tumor–bearing mice model across the indicated groups. Nuclei were counterstained with DAPI (blue). **Scale bar: 100 μm**. Label means **p* < 0.05, ***p* < 0.01, ****p* < 0.001, *****p* < 0.0001. Er: Erdafitinib.


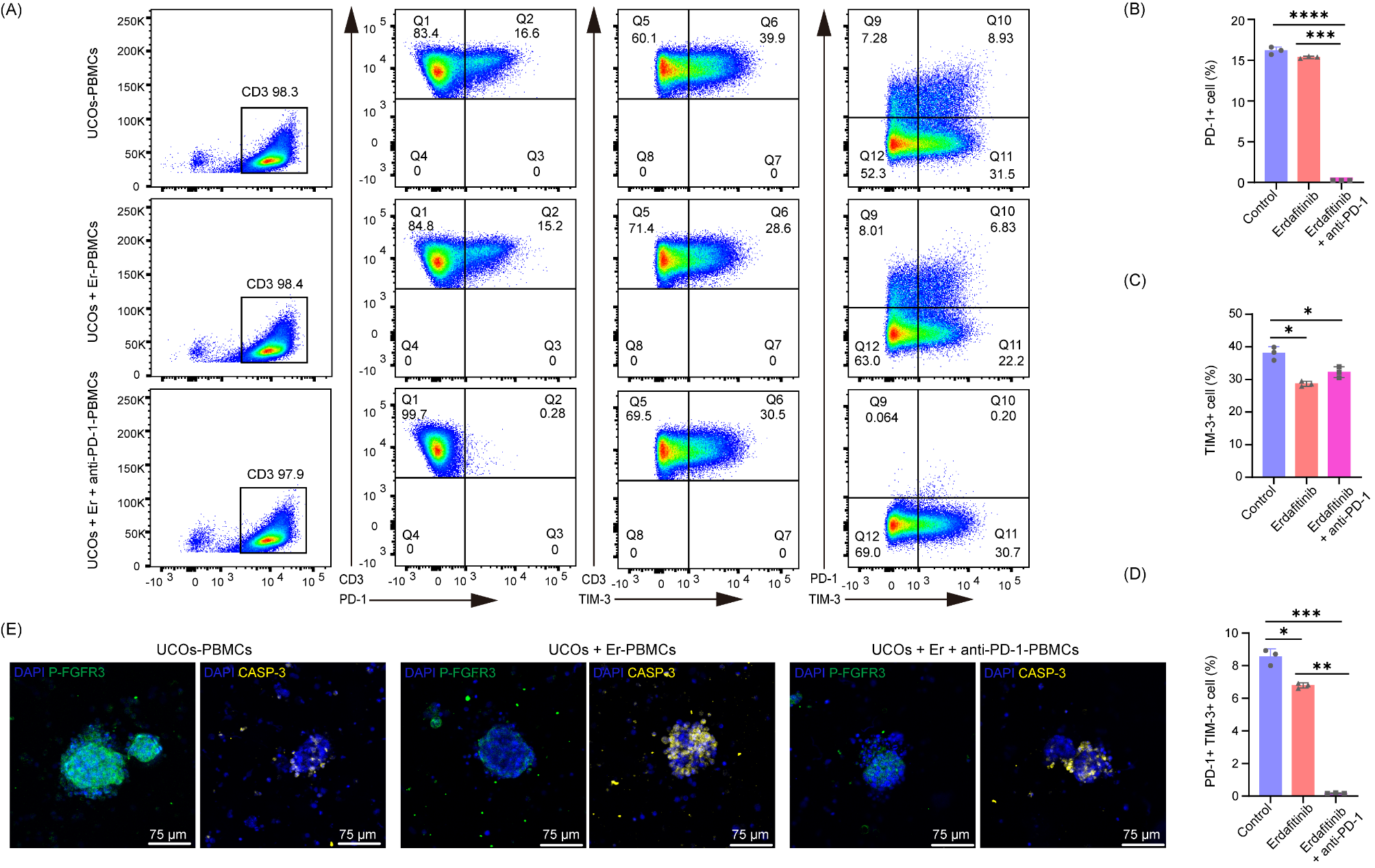


**Figure S8 Erdafitinib combined with anti-PD-1 immunotherapy reduces T-cell exhaustion and enhances cytotoxic function in co-culture system.** (A) Flow cytometry analysis of CD3^+^PD-1^+^, CD3^+^TIM-3^+^, and PD-1^+^TIM-3^+^ T cell proportions in different co-culture groups. (B–D) Flow cytometry analysis and statistical diagram showing the total proportion of PD-1^+^, TIM-3^+^, and PD-1^+^TIM-3^+^ T cells in different co-culture groups. (E) Immunofluorescence images of UCOs stained for P-FGFR3 and C-caspase3 in the indicated groups. Nuclei were counterstained with DAPI (blue). **Scale bar: 75 μm**. Label means **p* < 0.05, ***p* < 0.01, ****p* < 0.001, *****p* < 0.0001. Er: Erdafitinib.


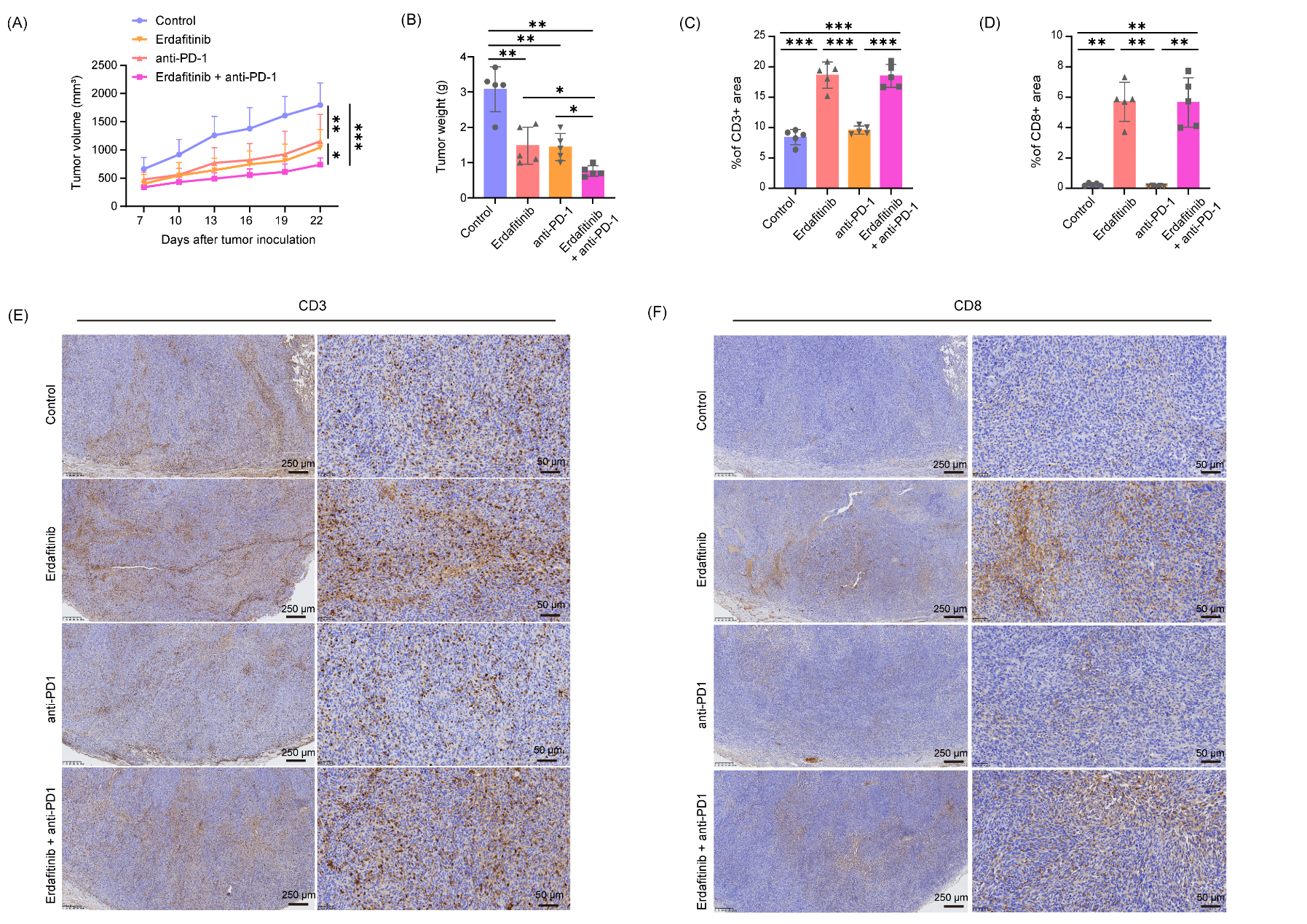


**Figure S9** **Combination of FGFR3 inhibition (erdafitinib) and anti-PD1 immunotherapy is synergistic in *FGFR3*–mutant UC.** (A–B) Tumor growth curves (A) and tumor weights (B) of the groups receiving control, erdafitinib, nivolumab, and combination therapy, respectively. Tumor volume = (Length × Width^2^)/2. (C–D) Statistical analysis of Immunohistochemistry (IHC) staining showed the infiltration of CD3^+^and CD8^+^ T cells within the indicated groups. (E–F) Immunohistochemistry (IHC) staining showed the CD3 (E) and CD8 (F) infiltration in the indicated groups from the mice model. **Scale bar: 250** **μm (left), 50 μm (right).** Label means **p* < 0.05, ***p* < 0.01, ****p* < 0.001, *****p* < 0.0001.
